# Supplementary figures and images for: Sodium hypochlorite inactivation of human CJD prions
Source: PLoS One. 2024 Nov 7;19(11):e0312837. doi: 10.1371/journal.pone.0312837 (PMC11542847; doi:10.1371/journal.pone.0312837)

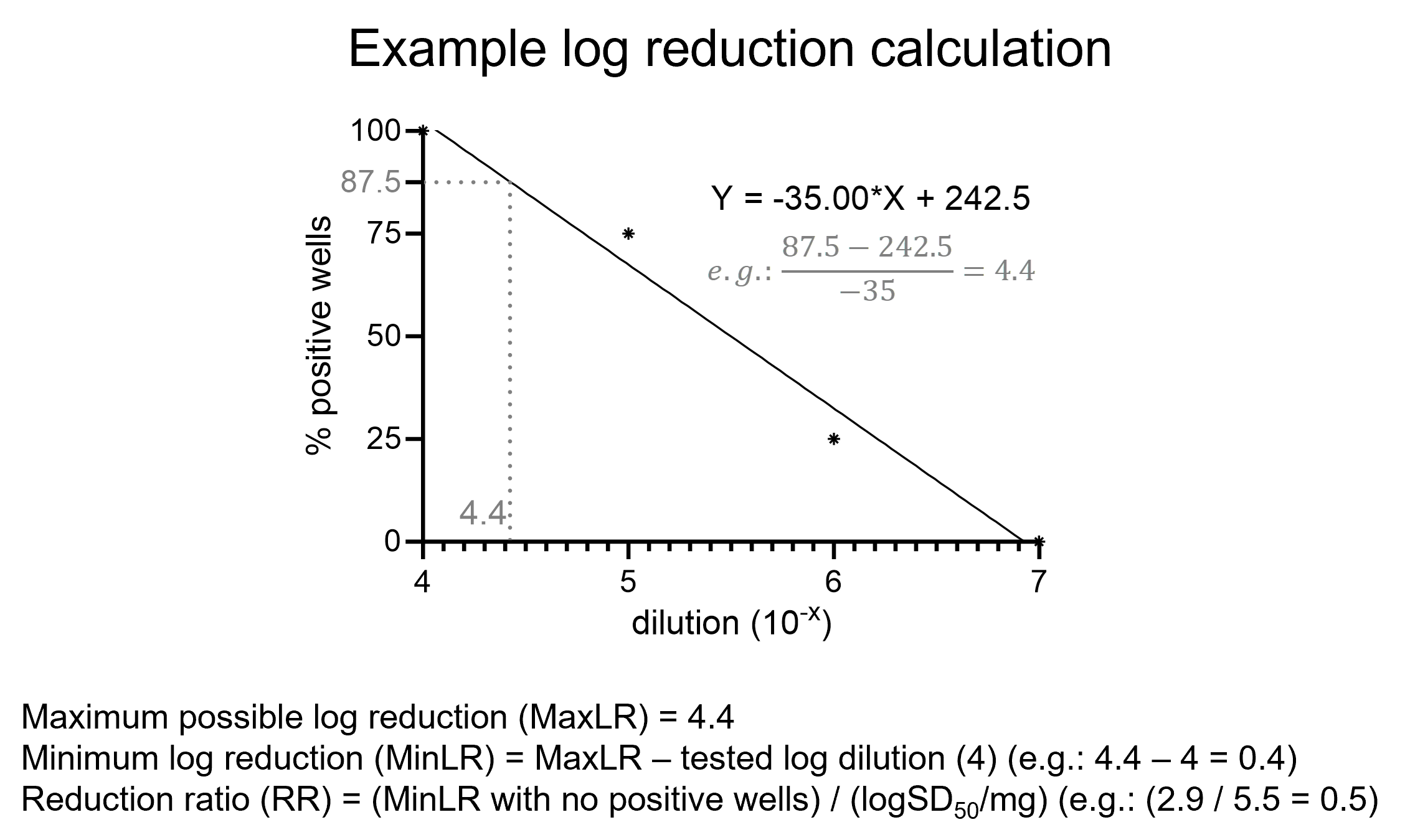

Supplement: S1 Fig — An example calculation is shown where the simple linear regression is drawn from the percent positive wells at each 10-fold dilution of an untreated brain homogenate. The equation is displayed above the curve. The example in gray is for a sample that following treatment displays 7 out of 8 positive wells (87.5% positive). Inserted into the equation this gives the equivalent of a 4.4 log reduction (MaxLR). However, since the sample was tested at a 4-log dilution, the possibility cannot be excluded that seeding activity might still be present at a lesser, untested dilution. Therefore, the minimum log reduction (MinLR) is calculated as the MaxLR minus the 4 logs that could not be tested. The actual log reduction for each treatment may fall at or between the MaxLR and MinLR. The log reduction ration (RR) is calculated as (MinLR with no positive wells) / (logSD50/mg brain tissue) to account for differences in starting seeding titer of the brain material (Tables 1 and 2). (TIF) [file pone.0312837.s001.tif]
